# Sclerosing mesenteritis mimicking metachronous peritoneal metastases from descending colon adenocarcinoma

**DOI:** 10.1186/s12957-017-1214-4

**Published:** 2017-08-01

**Authors:** Toshifumi Watanabe, Shiro Terai, Tomoya Tsukada, Masaki Takeshita, Koshi Matsui, Koji Amaya, Masahide Kaji, Kiichi Maeda, Koichi Shimizu, Junko Saito, Kentaro Mochizuki, Akio Uchiyama

**Affiliations:** Department of Surgery, Toyama Prefectural Central Hospital, Nishinagae 2-2-78 Toyama, Toyama, 9308550 Japan

**Keywords:** Colorectal cancer, Diagnosis, Peritoneal neoplasm, Positron emission tomography, Sclerosing mesenteritis, Surgery

## Abstract

**Background:**

Sclerosing mesenteritis is a non-neoplastic inflammatory disease that occurs in the bowel mesentery. Distinguishing sclerosing mesenteritis from neoplasms may be difficult because of the clinical and radiographic similarities between the two disease entities.

**Case presentation:**

We report a case of sclerosing mesenteritis mimicking peritoneal metastases of colorectal carcinoma. A 73-year-old man with stage II descending colon adenocarcinoma with poor prognostic features was found to have developed left lower abdominal quadrant masses on computed tomography (CT) 9 months after undergoing radical surgery. These masses were diagnosed as peritoneal metastases because they grew in size and displayed fluorodeoxyglucose (FDG) uptake 3 months later; thus, a laparotomy was performed. The masses, which were localized in the jejunal mesentery, were excised completely via segmental jejunal resection. Histopathological analysis confirmed that the masses were sclerosing mesenteritis. The patient showed no signs of sclerosing mesenteritis or colorectal carcinoma recurrence during follow-up.

**Conclusions:**

In patients suspected of having localized peritoneal metastasis from malignancies, any masses must be sampled by surgical excisional biopsy and subsequently examined to rule out alternative diagnoses, such as sclerosing mesenteritis.

## Background

Sclerosing mesenteritis is non-specific inflammatory mass-forming lesion in the mesenteric connective tissue that is characterized by variable degrees of fibrosis, chronic inflammation, and fat necrosis [1–5] and mainly affects the mesentery of the small bowel [2–9]. Although the exact etiology of sclerosing mesenteritis has not been elucidated [1–6, 9–14], it has been reported that the possible risk factors for the disease include malignancy, autoimmune disease, infection, ischemia, trauma, and a history of previous surgery [3, 5, 8, 11, 12]. Sclerosing mesenteritis is sometimes indistinguishable from neoplasms because its manifestations and radiographic findings may be identical to those of malignancies [3, 6, 7]. Despite these similarities, sclerosing mesenteritis is treated very differently than malignancies; thus, it is very important that sclerosing mesenteritis is diagnosed correctly so that the disease can be managed adequately.

Here, we report the case of a patient who underwent surgery for presumed metachronous localized peritoneal metastases from descending colon cancer and was ultimately diagnosed with sclerosing mesenteritis and thus avoided receiving unnecessary chemotherapy.

## Case presentation

**Fig. 1:**
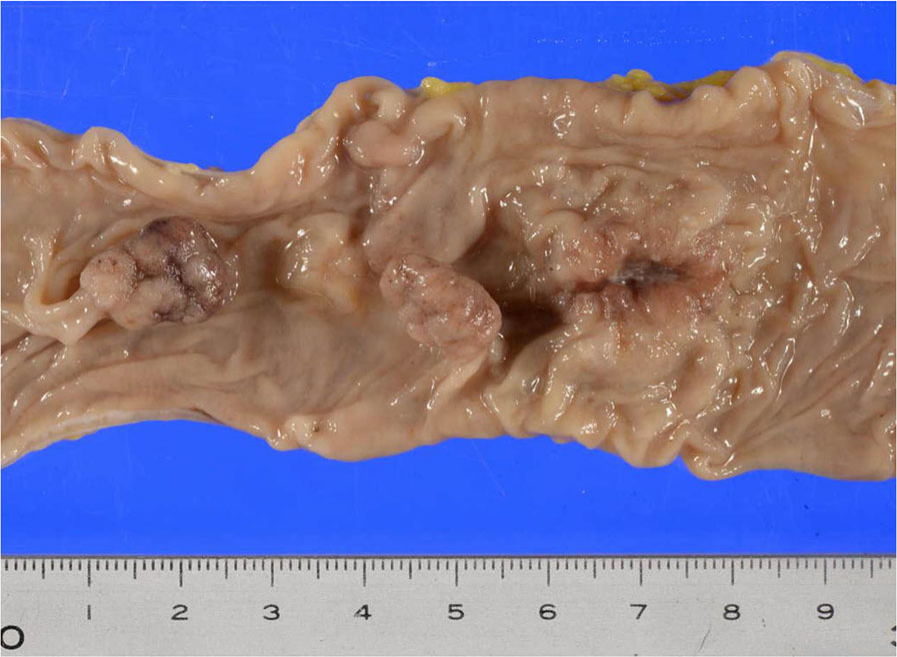
Histopathological analysis of a primary type II tumor in the descending colon. The pathological diagnosis was moderately differentiated tubular adenocarcinoma that had invaded the subserosa and was without lymph node metastasis. The tiny polyps were diagnosed as tubular adenoma

A 73-year-old man with advanced descending colon carcinoma was referred to our hospital for surgery. The patient had previously undergone a hemithyroidectomy for papillary thyroid carcinoma and an appendectomy for acute appendicitis. He was also receiving medications for the treatment of type 2 diabetes mellitus and the prevention of cerebral infarction. His carcinoembryonic antigen (CEA) and carbohydrate antigen (CA) 19-9 levels were 4.0 μg/L and 4.7 kU/L, respectively, and were thus each within their normal ranges. Computed tomography (CT) was negative for metastatic lesions. The patient underwent a laparoscopic partial colectomy and regional lymph node dissection. The pathological diagnosis was moderately differentiated tubular adenocarcinoma that had invaded the subserosa (T3) and was without lymph node metastasis (N0) (Fig. 1). The lymph node count was below 12. The patient was considered to have high-risk stage II colorectal cancer but elected not to undergo adjuvant chemotherapy. One month after surgery, he developed an adhesion-related small intestinal obstruction and underwent a partial jejunal resection.

**Fig. 2:**
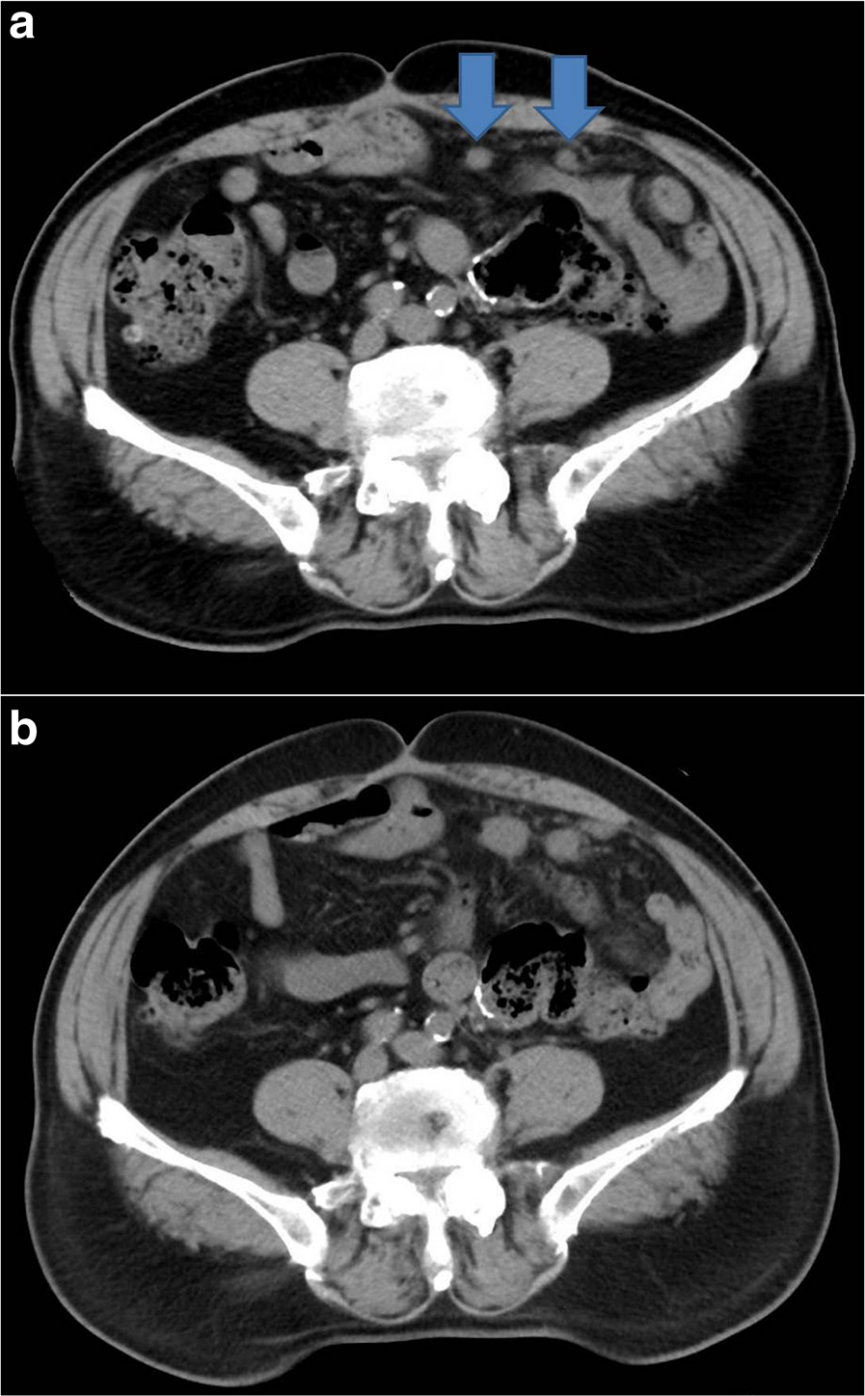
CT imaging. **a** Soft-tissue density masses arose in the left lower abdominal quadrant 9 months after radical surgery, as indicated by the *arrows*. **b** The masses grew in size during the ensuing 3 months

**Fig. 3:**
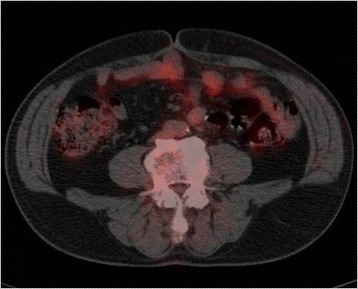
PET/CT imaging. PET/CT showing that the masses displayed increased FDG uptake

**Fig. 4:**
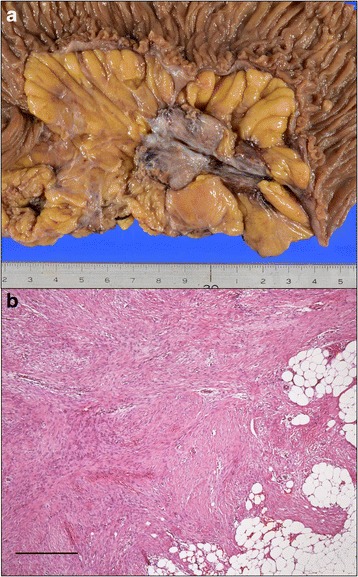
Gross and pathological findings (Hematoxylin-Eosion staining) pertaining to the resected specimens. **a** The mesenteric nodules were grayish white, well-delineated solid masses. **b** Collagen fibers and fibroblasts had proliferated and infiltrated the peripheral fatty tissues. The *scale bar* represents 500 μm

Nine months after the primary surgery, the patient underwent CT, which revealed soft tissue attenuating masses in the left lower abdominal quadrant (Fig. 2a). The patient did not have any symptoms, and his laboratory data, including his tumor marker levels, were within normal limits (his CEA and CA19–9 levels were 3.1 μg/L and 4.4 kU/L, respectively). The masses increased in size during the ensuing 3 months (Fig. 2b) and displayed increased fluorodeoxyglucose (FDG) uptake, with an SUV max of 2.9 on positron emission tomography (PET scan) (Fig. 3). The masses were attributed to peritoneal metastasis; thus, chemotherapy was recommended. However, the patient elected not to undergo chemotherapy because he was unwilling to allow his quality of life to adversely be affected by the therapy. He instead elected to undergo localized tumor excision; thus, a laparotomy was performed. Intraoperatively, the masses were found to be located in the mesentery of the jejunum. Complete excision of these mesenteric masses warranted resection of the affected intestinal segment because the vascular supply to the segment was severely compromised. Grossly, the resected nodules were grayish white, well-demarcated solid masses (Fig. 4a). Microscopically, the masses displayed collagen fiber and fibroblast proliferation and contained a few inflammatory cells. The fibroblasts had infiltrated and surrounded the peripheral fatty tissue. These findings were consistent with sclerosing mesenteritis (Fig. 4b).

The patient suffered from postoperative ileus but recovered with conservative treatment and showed no signs of sclerosing mesenteritis or descending colon cancer recurrence after 6 and 18 months of follow-up, respectively.

## Discussion

Sclerosing mesenteritis is an inflammatory process within the mesentery and can be categorized into the following three subgroups, depending on the predominant tissue component: mesenteric panniculitis, which is characterized by chronic inflammation; mesenteric lipodystrophy, which is characterized by fat necrosis; and retractile mesenteritis, which is characterized by fibrosis [2, 8, 10]. Distinguishing sclerosing mesenteritis from neoplasms is sometimes difficult because the disease can mimic lymphoma and digestive or gynecologic organ malignancies both clinically and radiographically [3, 6, 7]. Sclerosing mesenteritis remains asymptomatic in 30–50% of cases [1, 10]. The patient in the present case had no clinical manifestations of disease, nor did his laboratory studies exhibit specific abnormalities. The most common symptoms of the disease include fever, abdominal pain, abdominal mass, and weight loss [1–3, 5]; however, these symptoms are nonspecific and are associated with numerous other diseases [4]. Laboratory studies may show an elevated white blood cell count and/or erythrocyte sedimentation rate or anemia but are also unhelpful with respect to making a definitive diagnosis of the disease [4, 5].

The CT findings of sclerosing mesenteritis will vary depending on the predominant features of the disease [2]. Mesenteric panniculitis usually manifests as well-delineated masses composed of heterogeneous fatty tissues with increased densities [2, 11, 13]. In contrast, retractile mesenteritis presents as homogenous masses with a greater proportion of soft tissues [2, 13]. The fat ring sign, which signifies the preservation of a halo of fat around blood vessels, and the presence of a tumoral pseudocapsule consisting of a peripheral band of fatty tissue with soft tissue attenuation that protects normal mesentery from the inflammatory process, are both somewhat specific for mesenteric panniculitis [2, 10]. However, these two CT findings may disappear when mesenteric panniculitis evolves into retractile mesenteritis [2]. There are some reports indicating the superiority of magnetic resonance imaging (MRI) findings for diagnosing sclerosing mesenteritis [15, 16]. If we had performed MRI before laparotomy, the specific MRI findings might be provided. But it was difficult that the patient was diagnosed with no malignancy because the abdominal masses displayed rapid enlargement. The results of recently published studies indicate that PET scan findings enable clinicians to differentiate between benign and neoplastic mesenteric processes [9, 17]. In these studies, all patients whose mesenteric nodules displayed no FDG uptake had no malignant involvement of the mesentery. The authors of these studies concluded that negative PET had high diagnostic accuracy with respect to excluding tumoral mesenteric involvement [9, 17]. On the other hand, Ehrenpreis et al. [14, 18] reported that PET did not appear to be useful to distinguish an inflammatory mesenteric mass due to mesenteric panniculitis from a malignant mesenteric mass. Furthermore, false-positive results have been observed because some phases of the evolution of sclerosing mesenteritis present as hypermetabolic lesions [9, 17]. This was the case for the patient in the present study. If the patient had received chemotherapy for the diagnosis of peritoneal recurrence based on his radiographic findings alone, he would have received unnecessary treatment.

The prognosis of sclerosing mesenteritis is generally regarded as favorable with supportive treatment [1, 6, 7, 12]. In spite of having clinical and radiographic findings similar to those of malignancies, sclerosing mesenteritis is treated differently than malignancies. Radiological examinations, such as CT or PET scan, may be helpful tools for distinguishing sclerosing mesenteritis from alternative diagnoses, especially in patients suspected of having oncologic disease; however, it is necessary to determine whether malignant cells are present or absent to provide appropriate treatment for the disease. As some studies have demonstrated [4, 13], only complete histologic analyses of tissue samples obtained by surgery can rule out malignancy.

In conclusion, it is necessary to excise and examine mesenteric nodules in patients with a history of malignancy to distinguish tumor recurrence from alternative diagnoses, such as sclerosing mesenteritis.

## Conclusions

Sclerosing mesenteritis can mimic peritoneal metastasis from malignancies, and it is necessary to excise and examine a mesenteric nodule developing in patients with a history of malignancy to distinguish recurrence and differential diagnosis such as sclerosing mesenteritis.

## Abbreviations

CA19-9: Carbohydrate antigen
CEA: Carcinoembryonic antigen
CT: Computed tomography
FDG: Fluorodeoxyglucose
MRI: Magnetic resonance imaging
PET scan: Positron emission tomography
